# Inhibition of Hypoxia-Inducible Factor-1α (HIF-1α) Protein Synthesis by DNA Damage Inducing Agents

**DOI:** 10.1371/journal.pone.0010522

**Published:** 2010-05-07

**Authors:** Jessica Jie Wei Lou, Yee Liu Chua, Eng Hui Chew, Jie Gao, Martin Bushell, Thilo Hagen

**Affiliations:** 1 Department of Biochemistry, Yong Loo Lin School of Medicine, National University of Singapore, Singapore, Singapore; 2 School of Pharmacy, University of Nottingham, University Park, Nottingham, United Kingdom; University of Hong Kong, Hong Kong

## Abstract

Hypoxia-inducible factor (HIF) is a heterodimeric transcription factor that is composed of a hypoxia-inducible α subunit (HIF-1α and HIF-2α) and a constitutively expressed β subunit (HIF-1β). HIF mediates the adaptation of cells and tissues to low oxygen concentrations. It also plays an important role in tumorigenesis and constitutes an important therapeutic target in anti-tumor therapy. We have screened a number of reported HIF inhibitors for their effects on HIF-transcriptional activity and found that the DNA damage inducing agents camptothecin and mitomycin C produced the most robust effects. Camptothecin is a reported inhibitor of HIF-1α translation, while mitomycin C has been reported to induce p53-dependent HIF-1α degradation. In this study we demonstrate that the inhibitory effect of mitomycin C on HIF-1α protein expression is not dependent on p53 and protein degradation, but also involves HIF-1α translational regulation. Initiation of a DNA damage response with the small molecule p53 activator NSC-652287 (RITA) has been reported to inhibit HIF-1α protein synthesis by increasing the phosphorylation of eIF2α. However, we show here that even when eIF2α phosphorylation is prevented, the DNA damage inducing drugs mitomycin C, camptothecin and NSC-652287 still inhibit HIF-1α protein synthesis to the same extent. The inhibitory effects of camptothecin on HIF-1α expression but not that of mitomycin C and NSC-652287 were dependent on cyclin-dependent kinase activity. In conclusion, specific types of DNA damage can bring about selective inhibition of HIF-1α protein synthesis. Further characterization of the involved mechanisms may reveal important novel therapeutic targets.

## Introduction

During tumorigenesis, occurrence of hypoxia contributes to aggressive tumor progression, resistance to radiation and chemotherapy and poor prognosis. Hypoxia-inducible factor (HIF) is the key transcription factor that mediates the adaptation of cells and tissues to a hypoxic tumor environment. Its transcriptional targets include numerous genes involved in angiogenesis, erythropoiesis, glycolysis and cell proliferation [1], [2]. HIF is known to be upregulated in many human cancers, where it mediates the adaptation to the hypoxic tumor environment resulting from rapid tumor expansion that exceeds the development of new blood vessels. HIF has also been shown to directly promote tumorigenesis, for instance by inducing genetic instability via transcriptional downregulation of DNA mismatch repair proteins [3]. Furthermore, HIF has been reported to downregulate the intracellular adhesion molecule E-cadherin, thus contributing to loss of cell-cell adhesion in tumors [4]–[6], and to induce the expression of lysyl oxidase, thereby promoting tumor cell migration and metastasis [7]).

HIF is a heterodimer composed of a hypoxia-inducible α subunit (HIF-1α and HIF-2α) and a constitutively expressed β subunit (HIF-1β). It is regulated primarily through oxygen-dependent changes in the stability of the α subunit. Under normoxic conditions, HIFα is hydroxylated at two conserved proline residues (Pro402 and Pro564 in HIF-1α) by a family of oxygen- and 2-oxoglutarate-dependent prolyl 4-hydroxylases [8], [9]. Hydroxylated HIF-1α is recognized by the von Hippel-Lindau (pVHL) protein and rapidly ubiquitinated by the associated pVHL/Elongin B/C/Cul2 ubiquitin E3 ligase, followed by its degradation by the 26S proteasome. The low availability of oxygen under hypoxic conditions results in the inhibition of prolyl hydroxylase activity and consequently in the stabilization of HIF-1α protein. Upon nuclear translocation, HIF-1α forms a heterodimeric transcription factor with HIF-1β which binds to hypoxia-response elements and transactivates HIF target genes.

In addition to the oxygen-dependent posttranslational regulation, HIF-1α is also known to be regulated at various other levels, including gene transcription, protein translation and pVHL-independent protein degradation. For instance, insulin and growth factors such as insulin-like growth factor 1 and 2 and heregulin are known to increase HIF-1α protein concentrations by stimulating its protein synthesis in a 5′untranslated region (5′UTR)-dependent manner via activation of phosphatidylinositol 3-kinase, Akt and mTOR signaling [10], [11]. In addition, HIF-1α protein synthesis has been reported to be regulated by the RNA binding proteins HuR and PTB as well as via stress-induced phosphorylation of eIF2α [12]–[14]. There is also evidence for pathways that control HIF-1α stability in an oxygen-independent manner. For instance, Hsp90 inhibitors as well as the transcription factor FOXO-4 have been reported to induce the degradation of HIF-1α in a pVHL-independent manner [15]–[18].

Given the role of HIF in cancer, the development of HIF-inhibitory agents is of great importance. Search for HIF inhibitors and validation of their efficacy as anticancer agents is required. Indeed, a number of novel small molecule inhibitors of HIF have been identified through high-throughout screening of the National Cancer Institute (NCI) chemical repository or natural product-like combinatorial library [19]–[23]. In addition, various other agents have been found to have HIF inhibitory activity, however, the exact mechanism of action for most of these inhibitors remains unknown. Elucidation of the involved molecular mechanisms is critical to improve our understanding of the HIF signalling pathways and to allow the development of more specific and potent inhibitors. In this study, we have characterized and investigated the mechanism of action of a number of reported HIF inhibitors and identified the regulation of HIF-1α protein synthesis as an important target of several HIF-inhibitory compounds.

## Methods

### Cell lines, plasmid constructs, mutagenesis and transfection of HEK293 cells

All used cell lines were obtained from commercial sources (ATCC and Invitrogen). The expression vectors for wild type or P402A/P564A HIF-1α, carrying a C-terminal V5 or FLAG tag were as previously described [24]. Briefly, the HIF-1α coding sequence was ligated into pcDNA3 or pcDNA3.1/Hygro using the KpnI and XbaI sites, including a V5-tag immediately 5′ to the stop codon. For the 5′UTR containing HIF-1α plasmids, the 5′UTR (derived from IMAGE clone 3842146) was inserted into the NheI site of pcDNA3.1/Hygro and the internal XhoI site in the HIF-1α coding sequence. To generate the EGFP-HA-(HIF-1α-5′UTR) plasmid, the HIF-1α 5′UTR (from −260 to +132 bp) was inserted upstream of the EGFP full length coding sequence into pcDNA3. To generate the GADD34 C-terminal expression plasmid, the coding sequence corresponding to amino acids 263 to 674 of human GADD34 was introduced into modified pcDNA3.1 including an N-terminal FLAG tag. For retroviral expression of Bcl-x_L_, the Bcl-x_L_ coding sequence was introduced into the EcoRI and XhoI sites of the Puro-MaRX retroviral expression vector (a kind gift by David Beach, Institute of Cell and Molecular Science, London). Retrovirus was generated in 293-gag-pol cells and pseudotyped with VSV-G.

The T-Rex system (Invitrogen) was used to generate cell lines with tetracycline-inducible expression of dnUbc12-HA [25]. For transfections, sub-confluent cells were transfected using Genejuice (Novagen) according to the manufacturer's instructions.

### Hypoxia incubations

Hypoxia incubations were carried out at a controlled oxygen tension (1%) using a Pro-ox 110 oxygen controller and Pro-ox in vitro chamber (BioSpherix, Redfield, NY).

### Immunoblotting

For immunoblotting, cells were washed with ice-cold PBS and then lysed in triton X-100 containing lysis buffer, as previously described [26]. Lysates were pre-cleared by centrifugation before use for Western blotting. Equal amounts of protein from total lysates were used for Western blot analysis. The following antibodies were used: mouse monoclonal anti-HIF-1α (BD Biosciences), mouse monoclonal anti-GAPDH (US Biological), mouse monoclonal anti-α-tubulin (Molecular Probes), mouse monoclonal anti-β-catenin (BD Biosciences), mouse monoclonal anti-cdc6 (Santa Cruz), mouse monoclonal anti-Bcl-x_L_ (Santa Cruz), mouse monoclonal anti-GSK-3β (BD Biosciences), mouse monoclonal anti-p53 (Sigma), rabbit polyclonal anti-NRF2 (Santa Cruz), rabbit polyclonal anti-c-Myc (Santa Cruz), goat polyclonal anti-CAND1 (Santa Cruz), rabbit polyclonal anti-Cul2 (Zymed), goat polyclonal anti-Skp2 (Santa Cruz), mouse monoclonal anti-BRCA1 (Calbiochem), rabbit polyclonal anti-phospho-Ser209-eIF-4E (Cell Signaling), rabbit polyclonal anti-eIF-4E (Cell Signaling), rabbit polyclonal anti-phospho-Ser51-eIF2α (Cell Signaling), mouse monoclonal anti-eIF2α (Cell Signaling), mouse monoclonal anti-HuR (Santa Cruz), mouse monoclonal anti-PTB (Abcam), rabbit polyclonal anti-phospho-p70 S6 kinase (Cell Signaling), rabbit polyclonal anti-p70 S6 kinase (Cell Signaling), mouse monoclonal anti-phospho-Thr308-Akt (Cell Signaling), mouse monoclonal anti-phospho-Ser473-Akt (Cell Signaling), monoclonal anti-V5 (Serotec), monoclonal anti-FLAG M2 (Sigma), rat monoclonal anti-HA (clone 3F10) (Roche). All Western blot results shown are representative of at least two independent experiments.

### Immunofluorescence

Cells were plated on coverslips and after treatment, the cells were fixed with 4% formaldehyde for 15 min. The cells were permeabilized using PBS containing 0.1% Triton X-100 and blocked with PBS +0.05% Tween20+5% fetal bovine serum. 1∶1500 dilutions of both primary and secondary antibody were used. Subsequently, the coverslips were mounted onto glass slides with VectorShield mounting media containing DAPI to label nuclei and viewed using a fluorescent Leica DM IRB microscope equipped with a Leica HCX PL FLUOTAR 63x/1.25 oil objective.

### Northern blot analysis

Cells were cultured in 60 mm plates and after drug treatment for 10 to 12 hours, total RNA was isolated using TRIzol reagent (Invitrogen). Equal amounts of denatured RNA were separated using a formaldehyde/1% agarose gel, blotted onto Zeta-Probe blotting membranes (Bio-Rad) and then crosslinked to the blot in a UV Stratalinker (UVP, Upland, CA). The Northern blot was probed with an [γ-32^P^]dCTP – (3000 Ci/mMol, NEN, Boston, MA) labeled HIF1α DNA probe at 65°C overnight using Church hybridization solution (179.6 mM Na_2_HPO4, 91.25 mM NaH_2_PO4, 7% SDS), washed and exposed to the phosphorimager (FUJI).

### siRNA-mediated gene silencing

For siRNA transfections, RNAi Max Lipofectamine (Invitrogen) was used as transfection agent according to the manufacturer's instructions with the annealed predesigned siRNA duplexes (Integrated DNA Technologies) at a final concentrations of 20 nM.

### HRE-dependent luciferase reporter assays

HIF-1α reporter assays were carried out as previously described [24] using a pGL3-HRE plasmid that was kindly provided by Kaye Williams (The University of Manchester). Briefly, cells were cultured in 12 well plates. At approximately 50% confluency, cells were transfected with 0.15 µg pGL3-HRE and 0.1 µg pRL-CMV (Promega) or 0.15 µg empty pGL3 DNA per well for 24 hours. Cells were then incubated at 1% O_2_ for 6 hours in the presence of different inhibitors, and firefly and renilla luciferase activities were assayed using the Steady-Glo or Dual Luciferase Assay System (Promega). Each drug treatment was carried out in duplicates and results shown are representative of three independent experiments.

## Results and Discussion

### Camptothecin, mitomycin C and YC-1 robustly inhibit hypoxia-induced HIF transcriptional activity in HEK293 cells

**Figure 1:**
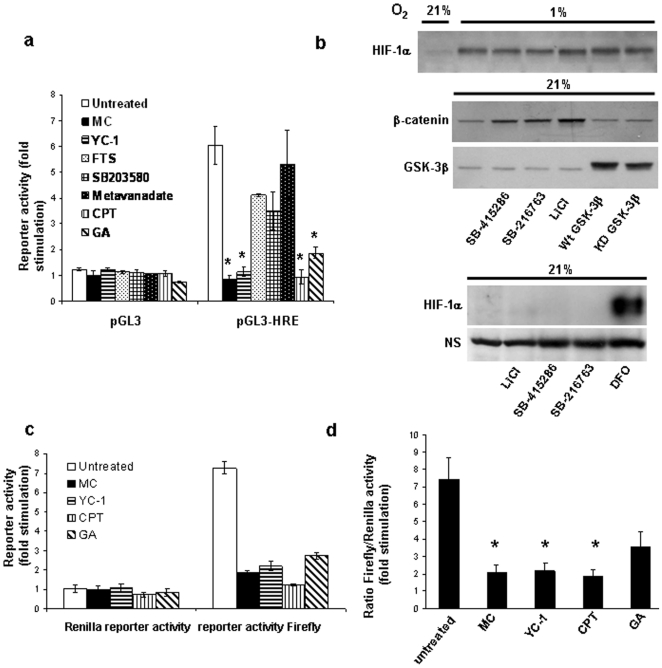
Effect of various HIF inhibitory compounds. (a) Hypoxia-dependent HIF-1α transcriptional activity was measured using HRE-dependent reporter assays as described under Materials and Methods. HEK293 cells were incubated at 1% O_2_ for 6 hours in the presence of the various inhibitors as indicated: mitomycin C (MC), 10 µg/ml; YC-1, 50 µM; S-trans,trans-farnesylthiosalicylic acid (FTS), 70 µM; SB203580, 20 µM; metavanadate, 50 µM; camptothecin (CPT), 2 µM; geldanamycin (GA), 10 µM. (a) Luminescence was measured and fold stimulation was obtained by normalizing the relative luciferase activity of cells cultured under hypoxic conditions to those of nontreated cells cultured under normoxic conditions. Results represent mean ± SEM of three independent experiments. Student's *t* test analysis of the ratio of fold stimulation obtained for cells transfected with pGL3-HRE to those transfected with the promoter-less pGL3 empty vector showed that treatment with MC, YC-1, CPT and GA significantly (*p<*0.05, denoted as an asterisk *, n = 3) reduced luciferase activity compared to untreated control. (b) HEK293 cells were transfected for two days with wild type or kinase-dead (K85R) GSK-3β expression plasmids, or treated with 20 µM SB-415286, 5 µM SB-216763, or 30 mM LiCl for 6 hours. In the upper panel, the cells were incubated at 1% O_2_ during the drug exposure. Cytosolic β-catenin concentrations were measured by lysing cells in hypotonic lysis buffer as previously described [26] In the bottom panel, 200 µM desferrioxamine (DFO) was used as a positive control for normoxic HIF-1α induction and a HIF-1α reactive non-specific (NS) band was used to demonstrate equal protein loading. (c,d) To study the effects of mitomycin C, YC-1, camptothecin and geldanamycin on HIF transcriptional activity in more detail, firefly and renilla luciferase activity of cells cotransfected with pGL3-HRE and pRL-CMV and incubated under hypoxic condition in the presence of MC, YC-1, CPT and GA (as in Fig. 1a) was measured. The renilla and firefly luciferase activities are shown in (c), the ratio of firefly to renilla luciferase activity in (d). The results are expressed as fold stimulation relative to the activity in nontreated cells under normoxic conditions. Statistical significance (p<0.05, n = 3) is indicated with an asterisk in the bottom panel.

Using a hypoxia-responsive luciferase reporter assay, we initially screened a number of reported HIF-inhibitory compounds [15], [16], [27]–[33] for their effects on HIF-transcriptional activity. To rule out the possibility of non-specific inhibitory effects, the luciferase activity of cells transfected with a pGL3 plasmid lacking the hypoxia response element (HRE) was also determined. While hypoxic incubation of cells transfected with pGL3-HRE resulted in an approximately six-fold stimulation of luciferase activity compared to cells incubated under normoxic conditions, no significant change was observed in cells transfected with empty pGL3 (Fig. 1a). Between the non-treated and each drug treatment group, statistical comparison (Student's *t* test) of the ratio of fold stimulation obtained for cells transfected with pGL3-HRE to those transfected with pGL3 empty vector showed that the guanylyl cyclase activator YC-1, the topoisomerase I inhibitor camptothecin, the DNA crosslinking agent mitomycin C, and the Hsp90 inhibitor geldanamycin significantly reduced (*p*<0.05) hypoxia-dependent HIF-transcriptional activity (Fig. 1a). In contrast, metavanadate, the p38 inhibitor SB203580 and the Ras inhibitor *S*-*trans,trans*-farnesylthiosalicylic acid did not produce a significant (*p*>0.05) reduction in luciferase activity.

We also investigated the role of glycogen synthase kinase-3, which has previously been reported to mediate destabilization of HIF-1α, thus leading to inhibition of HIF-1α dependent transcriptional activity [34]. SB-415286 and SB-216763, two selective GSK-3 inhibitors, did not increase HIF transcriptional activity in hypoxia, but actually led to a decrease in activity by 27% and 16%, respectively (n = 3). This decrease was at least partially due to non-specific drug effects as we also observed a reduction in the luciferase activity of cells transfected with empty control vector (14% and 11% for SB-415286 and SB-216763, respectively). As shown in Fig. 1b, the GSK-3 inhibitors SB-415263, SB-216763 and lithium chloride, while leading to the expected upregulation of cytosolic β-catenin (a GSK-3 substrate which is normally targeted for proteasome-dependent degradation upon phosphorylation), also had no significant effect on the hypoxia-induced HIF-1α protein stabilization. The inhibitors also did not affect normoxic HIF-1α protein concentrations. Furthermore, overexpression of wild type or dominant-negative GSK-3β at significant levels was without effect on HIF-1α protein abundance in hypoxia, arguing against a direct role of GSK-3 in regulating HIF-1α. In support of our results, a recent kinome screen for modulators of HIF activity did not identify GSK-3 [35]. Thus, GSK-3 is likely not a universal regulator of HIF-1α protein stability but may play a regulatory role in a cell type and tissue specific manner [34].

To further confirm that the effect of the most potent HIF-inhibitory agents mitomycin C, YC-1, camptothecin and geldanamycin was not due to non-specific effects or loss of cell viability, cells were cotransfected with the pGL3-HRE reporter construct and a Renilla luciferase control plasmid. As shown in Fig. 1c (upper panel), the four inhibitors had no significant effect on Renilla luciferase activity (statistical comparison of each treatment group with the nontreated group yielded *p* values >0.05), indicating that their effects are due to inhibition of HIF-transcriptional activity. When determining the ratio of HRE-dependent firefly luciferase activity over the activity of the constitutively expressed Renilla luciferase, the inhibitory effects of mitomycin C, YC-1 and camptothecin were still highly significant (Fig. 1c, lower panel, *p*<0.05). In contrast, inhibition by geldanamycin was no longer statistically significant (*p* = 0.06). We therefore focused on mitomycin C, camptothecin and YC-1 in further studies.

### Mitomycin C, camptothecin and YC-1 inhibit HIF-1α protein accumulation

**Figure 2:**
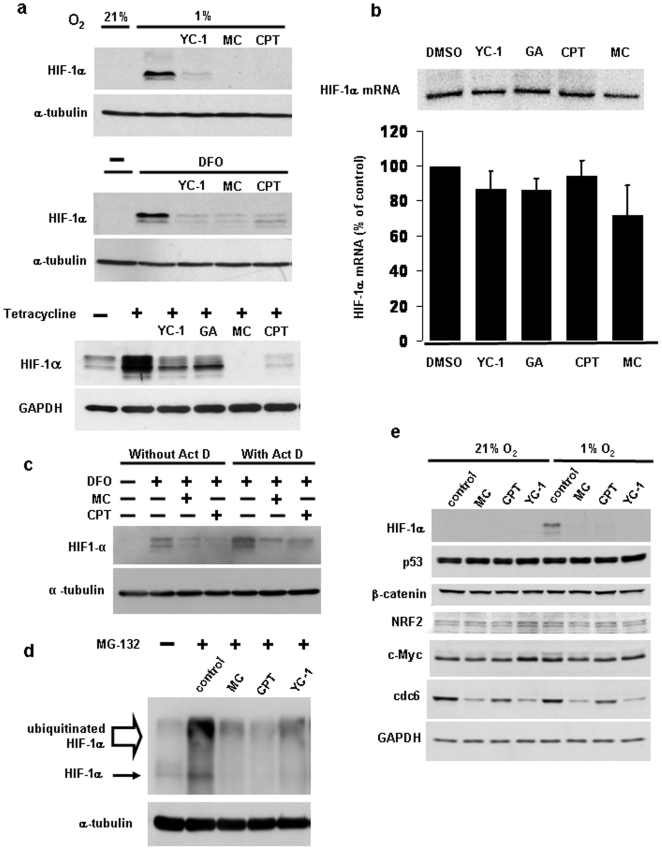
The effect of the mitomycin C, camptothecin and YC-1 is due to inhibition of HIF-1α protein synthesis. (a–b) HEK293 cells were incubated for 10 hours in the presence of YC-1 (50 µM), mitomycin C (10 µg/ml), camptothecin (2 µM), and geldanamycin (10 µM) and cell lysates subjected to Western blotting using the indicated antibodies. Cells were incubated at 1% oxygen during the last three hours of inhibitor treatment (upper panel) or in the presence of 200 µM desferrioxamine (DFO) during the inhibitor treatment for 10 hours (middle panel), as indicated. In the bottom panel, stably transfected cells with tetracycline inducible expression of dnUbc12 (5) were treated with 1 µg/ml tetracycline to block VHL mediated HIF-1α degradation for 9 hours and cotreated with the indicated drugs for the last 5 hours, followed by Western blotting. The presence of drugs during the induction time is indicated. (b) HIF-1α mRNA concentrations after inhibitor treatment for 10 to 12 hours were measured as described under Material and Methods. A representative autoradiogram is shown in the top panel. The bottom panel shows the average mRNA levels for each inhibitor treatment expressed relative to those of DMSO control, as determined by densitometry of autoradiograms obtained from three independent experiments. Statistical analysis using one-way ANOVA test across all treatment groups showed that all inhibitors exerted an insignificant (*p*>0.05, n = 3) effect. (c) Cells were treated with 200 µM desferrioxamine and the indicated drugs in the absence or presence of 5 µM actinomycin D for 8 hours, followed by Western blotting. (d) HIF-1α protein accumulation was measured by Western blotting after cotreatment of cells with 25 µM MG-132 (added to cells for the last 4 hours) and the various inhibitors as indicated (added to cells for the last 9 hours). (e) To confirm the specificity of the effect of mitomycin C, YC-1 and camptothecin, Western blotting for the indicated proteins was carried out after drug treatment of cells for 11 hours and incubation at 1% oxygen during the last 4 hours, as indicated. Inhibitor concentrations in (b–e) were as in (a).

To investigate the mechanism of action of the three more potent HIF inhibitors, we determined whether the effects on hypoxia-induced HIF transcriptional activity are due to inhibition of hypoxia-dependent HIF-1α protein accumulation. To this end, HIF-1α protein was measured in HEK293 cells exposed to 1% oxygen in the absence or presence of the three inhibitors. As shown in Fig. 2a, all three compounds markedly reduced the HIF-1α protein accumulation in hypoxia. Similarly, the compounds also inhibited HIF-1α protein accumulation induced by prolyl hydroxylase inhibition with desferrioxamine. All three drugs also strongly inhibited HIF-1α protein when oxygen- and pVHL-dependent HIF-1α ubiquitination was blocked (Fig. 2a). This was achieved using a previously described cell line with tetracycline-inducible expression of dominant-negative Ubc12, which results in inhibition of all cullin E3 ubiquitin ligases, including the pVHL/Elongin B/C/Cul2 E3 ligase [25]. The finding that YC-1, mitomycin C and camptothecin could reduce HIF-1α protein concentrations when prolyl hydroxylase- and pVHL-mediated HIF-1α degradation was blocked suggests that these compounds either inhibit HIF-1α mRNA or protein synthesis or that they induce HIF-1α protein degradation that is independent of the prolyl hydroxylase- and pVHL-mediated pathway.

### All three compounds specifically inhibit protein synthesis of HIF-1α

To determine the effects of the inhibitors on HIF-1α mRNA synthesis and stability, we carried out Northern blot analyses. As shown in Fig. 2b, YC-1 and camptothecin had no or only small effects on the HIF-1α mRNA concentration, while mitomycin C caused a 30% reduction in mRNA levels. However, statistical analysis using a one-way ANOVA test showed that this reduction was not significant (*p*>0.05). Also, a 30% reduction in HIF-1α mRNA is unlikely to account for the much greater mitomycin C-induced reduction in the HIF-1α protein concentration. In order to confirm that the effect of mitomycin C is independent of transcriptional downregulation of HIF-1α, we used actinomycin D to block transcription. Addition of actinomycin D to cells caused a paradoxical increase in the HIF-1α protein concentration (Fig. 2c), consistent with a previous report [36]. Mitomycin C caused proportionally the same decrease in HIF-1α protein expression in the presence or absence of actinomycin D, suggesting that its effect is independent of transcriptional regulation (Fig. 2c). Actinomycin D partially blocked the HIF-1α inhibitory effect of camptothecin.

To determine the effects on HIF-1α protein synthesis, cells were incubated in the presence of the 26S proteasome inhibitor MG-132. Under these conditions, HIF-1α protein degradation that is mediated through the prolyl hydroxylase and pVHL dependent as well as through alternative mechanisms would be inhibited. Mitomycin C, camptothecin and YC-1 markedly reduced the MG-132-induced accumulation of both the 120 kDa HIF-1α protein as well as high molecular weight ubiquitinated HIF-1α protein (Fig. 2d), indicating that these agents inhibit the synthesis of new HIF-1α protein.

To confirm that the drug-induced decrease in the HIF-1α protein abundance is specific, we first determined the effect of mitomycin C, camptothecin and YC-1 on the steady state concentrations of a number of other short lived cellular proteins under normoxic and hypoxic conditions (Fig. 2e). The drugs were without effect on the abundance of p53, β-catenin, NRF2, c-Myc, and the loading control GAPDH. However, we found that cdc6 protein abundance was reduced by mitomycin C and YC-1, and to a lesser degree by camptothecin. Given that the effect of the drugs on the cdc6 protein was prevented by addition of 26S proteasome inhibitor (data not shown), it appears likely that they affect the protein via a different mechanism. We also estimated the effects of the drugs on general protein synthesis by measuring incorporation of L-[^35^S]-methionine/L-[^35^S]-cysteine into cellular proteins. As shown in Supplementary Fig. S1, both camptothecin and mitomycin C slightly inhibited overall protein translation. However, this relatively small inhibitory effect is unlikely to account for the marked reduction in HIF-1α protein expression observed with both drugs.

Taken together, the results presented suggest that mitomycin C, camptothecin and YC-1 are relatively selective inhibitors of HIF-1α. All three drugs inhibit HIF-1α via effects on protein synthesis. Our finding of camptothecin-induced inhibition of HIF-1α protein synthesis agrees with previous studies [28], [29]. In contrast, mitomycin C was reported by Kaluzova *et al.* [33] to induce p53-dependent degradation of HIF-1α, while different mechanisms have been proposed for the action of YC-1. In further studies, we focused on the mechanism of action of mitomycin C- and camptothecin-mediated HIF inhibition.

### Inhibition of HIF-1α protein accumulation by mitomycin C is not due to an effect on protein degradation

Given that our conclusion with regards to mitomycin C-mediated inhibition of HIF-1α protein accumulation contradicts a previous study, we wanted to confirm that protein degradation is not involved in the drug effect. We first determined the dose and time dependence with which mitomycin C inhibited HIF-1α protein expression (Supplementary Fig. S2a). Mitomycin C caused a dose-dependent decrease in desferrioxamine-induced HIF-1α protein expression, with a maximum effect observed at a concentration of 10 µg/ml. A similar dose dependence for mitomycin C-induced HIF-1α inhibition was observed under hypoxic conditions (Supplementary Fig. S2b). To determine the time dependence of the drug effect of mitomycin C, cells were treated with desferrioxamine, which caused a time-dependent increase in HIF-1α protein expression with a maximal effect at approximately 10 hours (Supplementary Fig. S2c). Three hours after desferrioxamine addition, mitomycin C was added and the HIF-1α protein started to decrease gradually over time compared to the control starting from 4 hours. At 10 hours of mitomycin C treatment, HIF-1α decreased to non-induced control levels. In all further experiments, we therefore used mitomycin C at a concentration of 10 µg/ml for 8 to 12 hours.

**Figure 3:**
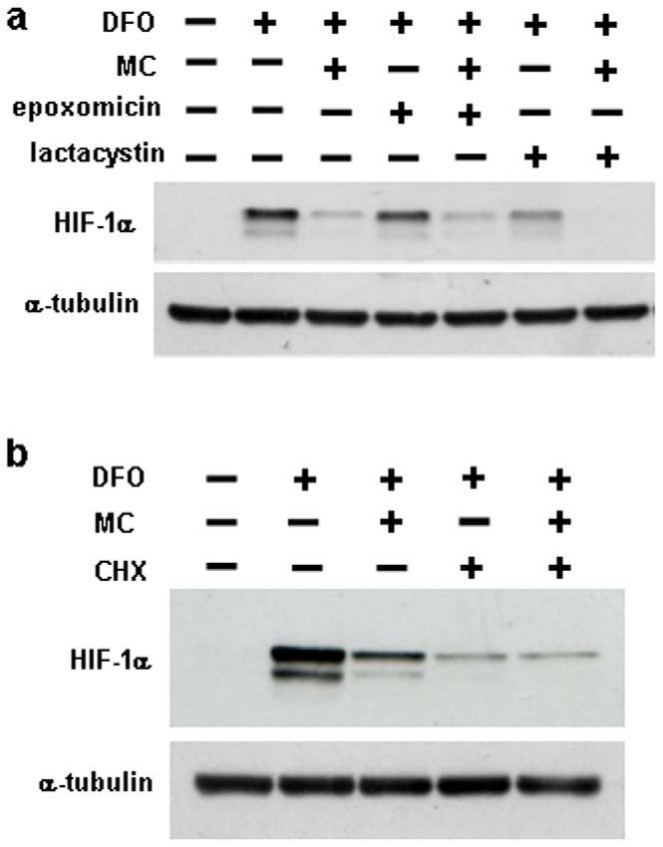
Inhibition of HIF-1α protein accumulation by mitomycin C is not due to an effect on protein degradation. (a) HEK293 cells were treated with 200 µM desferrioxamine (DFO) and 10 µg/ml mitomycin C for 10.5 hours, as indicated, in the absence or presence of the 26S proteasome inhibitors epoxomicin (1 µM) or lactacystin (5 µM). (b) The cells were pretreated with 200 µM desferrioxamine for three hours, followed by addition of 10 µg/ml mitomycin and 40 µM cycloheximide for 8 hours.

To study the involvement of the 26S proteasome in mitomycin C mediated HIF-1α inhibition, we used two specific inhibitors of proteasomal protein degradation, epoxomicin and lactacystin. Both drugs had no effect on the inhibition of desferrioxamine-induced HIF-1α accumulation by mitomycin C (Fig. 3a). Similarly, the proteasome inhibitor MG-132 and the inhibitors of lysosomal protein degradation, NH_4_Cl, chloroquine and bafilomycin were without effect (data not shown). When using a HIF-1α protein which was tagged with a FLAG- and a V5-tag at the N- and C-terminus, we observed no cleavage products when probed with either FLAG or V5 antibody (data not shown), indicating that the effect of mitomycin C was not due to proteolytic cleavage of HIF-1α protein. Consistent with this result, the protease inhibitors calpeptin, pepstatin A and E-64 did not inhibit the mitomycin-induced downregulation of endogenous HIF-1α (Supplementary Fig. S3a). We also did not observe accumulation of HIF-1α in the triton X-100-insoluble fraction upon treatment with mitomycin C (or camptothecin) (Supplementary Fig. S3b). Thus, our data suggest that inhibition of HIF-1α protein expression by mitomycin C is not due to degradation by the 26S proteasome, lysosome or other proteases or due to accumulation in insoluble aggregates. It has been reported that in the presence of proteasome inhibitor, mitomycin C decreases HIF-1α protein in the soluble fraction because the protein becomes insoluble and accumulates in the triton-insoluble fraction [33]. However, when using desferrioxamine to induce HIF-1α protein in HEK293 cells, we did not observe any accumulation of HIF-1α protein in the triton-insoluble fraction upon adding proteasome inhibitor MG-132, in the presence or absence of mitomycin C (data not shown).

We also tested whether mitomycin C still reduces HIF-1α protein concentrations if new protein synthesis is inhibited with cycloheximide. Addition of cycloheximide for 8 hours resulted in a significant reduction in desferrioxamine-induced HIF-1α protein (Fig. 3b), suggesting that HIF-1α still undergoes degradation in the presence of the iron chelator. Mitomycin C did not further reduce the HIF-1α protein abundance in the presence of cycloheximide, further suggesting that the compound does not induce HIF-1α degradation. It would be possible that mitomycin C-dependent HIF-1α requires new transcription and/or translation (e.g. transcriptional upregulation of genes that mediate HIF-1α degradation). However, the lack of effect of actinomycin D on mitomycin C-mediated HIF-1α inhibition (see Fig. 2c) argues against such a mechanism.

### Inhibition of HIF-1α protein expression by mitomycin C, but not by camptothecin is cell type dependent

**Figure 4:**
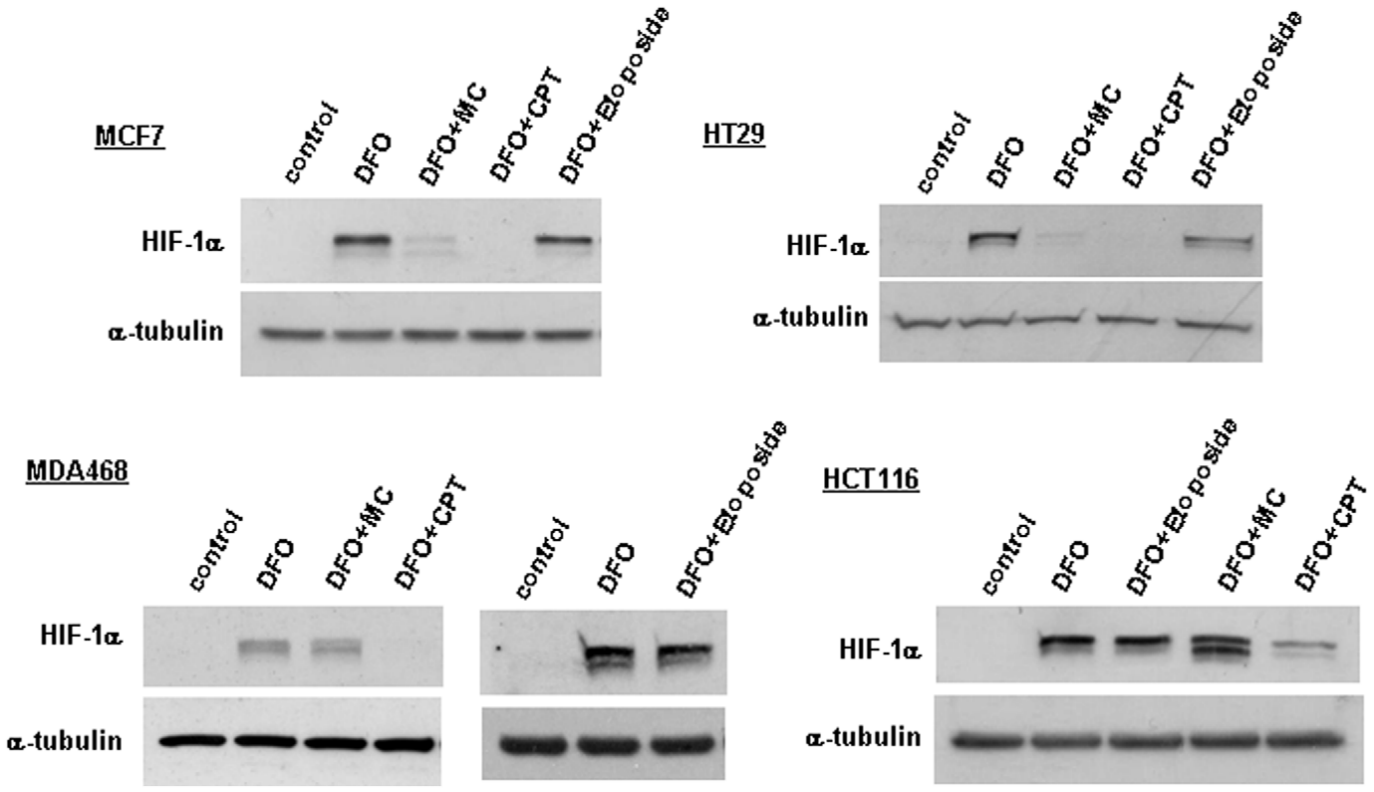
The effect of mitomycin C, camptothecin and etoposide on HIF-1α protein concentrations in different cell lines. The indicated cell types were treated with 200 µM desferrioxamine, 10 µg/ml mitomycin, 2 µM camptothecin and 50 µM etoposide, as specified, for 10 to 14 hours.

We next tested the effect of mitomycin C and camptothecin on desferrioxamine-induced HIF-1α protein expression in a number of additional cell lines. Camptothecin inhibited HIF-1α expression in all tested cell lines (Fig. 4). In contrast, mitomycin C was active in the breast cancer cell line MCF7 and the colon cancer cell line HT29, but not in MDA468 breast cancer and HCT116 colon cancer cells. These results suggest that the effect of mitomycin C, but not of camptothecin, is cell type dependent. When measuring the cytotoxicity of mitomycin C using MTT assays, the drug exhibited very similar potency in all four cell lines (data not shown), indicating that the differential effect is not due to toxicity or induction of apoptosis.

### Mitomycin C-mediated HIF-1α degradation does not require p53 transcriptional activity and is independent of cellular apoptosis

**Figure 5:**
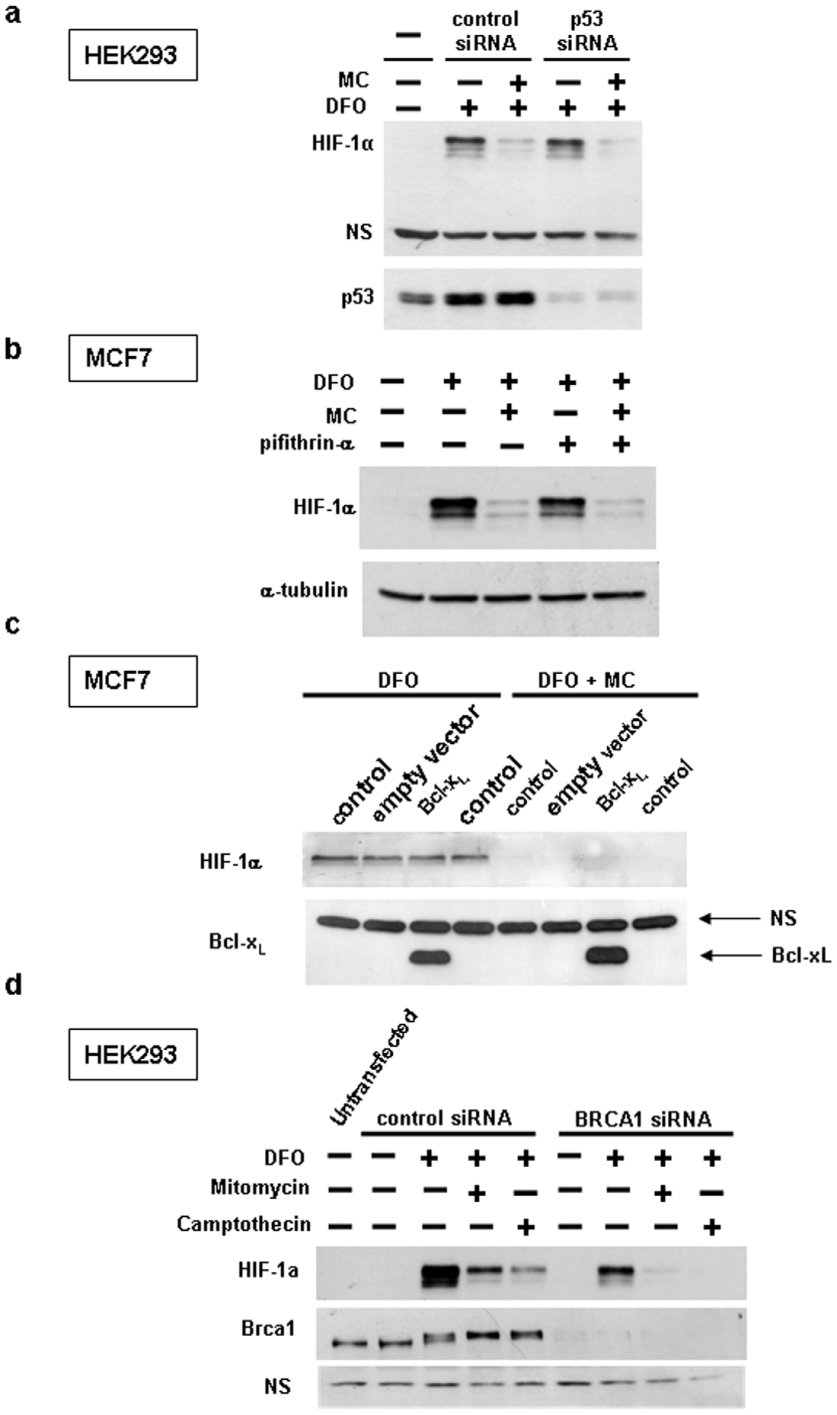
Inhibition of HIF-1α by mitomycin C is independent of p53 and DNA damage-induced apoptosis. (a) HEK293 cells were transfected with negative control or p53 siRNA for three days, followed by treatement with 200 µM desferrioxamine and 10 µg/ml mitomycin C for 10 hours and Western blotting of cell lysates with the indicated antibodies. (b,c) MCF7 cells were treated with 200 µM desferrioxamine and 10 µg/ml mitomycin C for 14 hours as indicated, followed by Western blotting of cell lysates. In (b) pifithrin-α (25 µM) was included, as indicated. In (c) MCF7 cells were retrovirally transduced with Bcl-x_L_ or empty vector, as indicated. (d) Brca1 was knocked down using siRNA for three days, as described under Materials and Methods, followed by drug treatment (200 µM desferrioxamine, 10 µg/ml mitomycin, 2 µM camptothecin), as indicated.

Mitomycin C induced inhibition of HIF-1α protein expression has been reported to be p53 dependent [33]. In contrast, our results in HEK293 cells, in which p53 is inhibited due to the constitutive expression of adenovirus proteins E1A and E1B [37]–[40] suggest that the effect of mitomycin C does not require p53-transcriptional activity. Furthermore, siRNA mediated silencing of p53 in HEK293 cells did not affect mitomycin C induced inhibition of HIF-1α expression (Fig. 5a). In addition, the inhibitory activity of mitomycin C towards HIF-1α protein expression in the different cell lines used in Fig. 4 does not correlate with their p53 status. Thus, mitomycin C was active in MCF7 cells with wild type p53 and HT29 cells with mutant p53, but was inactive in HCT116 cells with wild type p53 and MDA468 with mutant p53. Finally, addition of pifithrin-α, a small molecule inhibitor of p53 transcriptional activity, to wild type p53 expressing MCF7 cells did not affect HIF-1α inhibition by mitomycin C (Fig. 5b).

The finding that camptothecin inhibited HIF-1α protein expression in all cell lines indicates that its effect is also p53 independent. This is further supported by our finding that etoposide, a DNA damaging agent with a related mechanism of action to camptothecin (i.e. inhibition of topoisolerase II versus I) which activates p53 via a similar DNA damage response, did not affect HIF-1α protein expression (Fig. 4).

Mitomycin C induces cellular apoptosis via the intrinsic pathway. To determine if mitomycin C induced HIF-1α inhibition is a consequence of cellular apoptosis, a commonly used approach would be to block the intrinsic apoptotic pathway by overexpression of antiapoptotic Bcl-2 family members. However, paradoxically, some Bcl-2 family members are known to have proapoptotic effects in HEK293 cells. We therefore used MCF7 breast cancer cells and overexpressed Bcl-x_L_. Retroviral transduction of Bcl-x_L_ into MCF-7 cells resulted in a pronounced protein overexpression (Fig. 5c) and had a marked protective effect on mitomycin C induced apoptosis (data not shown). However, inhibiting apoptosis was without effect on mitomycin C mediated HIF-1α inhibition (Fig. 5c), suggesting that the drug effect is not a consequence of cellular apoptosis.

BRCA1 is a major mediator of cellular DNA damage repair response downstream of the ATM, ATR and checkpoint kinases. BRCA1 mutant cells have a 100 fold increased sensitivity to mitomycin C [41], emphasizing the importance of BRCA1 in the cellular response to the DNA crosslinking agent. We therefore tested the involvement of BRCA1-mediated signalling events for the effect of mitomycin C on HIF-1α protein expression by siRNA mediated silencing of BRCA1 in HEK293 cells. As shown in Fig. 5d, more than 90% knockdown of BRCA1 protein expression was achieved. Although BRCA1 knockdown reduced the basal expression of HIF-1α in the presence of desferrioxamine, it did not prevent the inhibitory effect of mitomycin C (as well as that of camptothecin). These results suggest that mitomcyin C induced inhibition of HIF-1α protein synthesis is independent of a full DNA damage response and may be mediated by signaling mediators upstream from BRCA1 (e.g. through DNA damage sensor and checkpoint kinases such as ATM, ATR and Chk2) or alternative pathways.

### Characterization of the effects of mitomycin C and camptothecin on protein synthesis regulatory pathways

**Figure 6:**
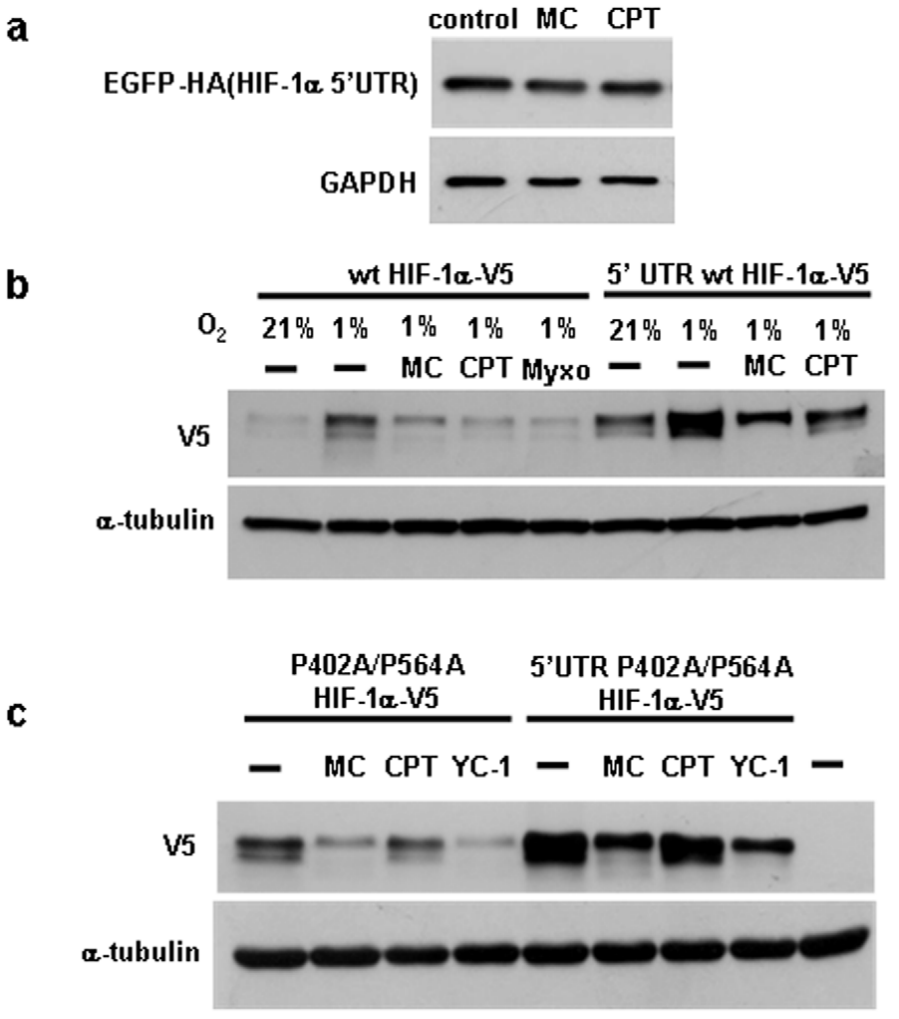
Mitomycin C and camptothecin mediated inhibition of HIF-1α protein synthesis is independent of the HIF-1α 5′UTR. (a) HEK293 cells were transfected with HIF-1α-5′UTR-EGFP-HA plasmid for two days and then treated with 10 µg/ml mitomycin, 2 µM camptothecin, followed by Western blotting with HA antibody. (b,c) Cells were transfected with the indicated plasmids and treated with the indicated drugs. Myxothiazol, a mitochondrial complex III inhibitor and well established inhibitor of HIF-1α served as positive control in (b).

Protein synthesis is known to be regulated at multiple levels in response to different signals and various types of cellular stress. HIF-1α has been reported to be regulated at the level of protein translation. For instance, insulin and growth factors are known to increase HIF-1α protein synthesis [10], [11]. Although the exact mechanisms involved are currently not clear, the regulatory effect is dependent on the HIF-1α 5′untranslated region (5′UTR) [10]. To test for the role of the 5′UTR in mediating the inhibitory effect of mitomycin C and camptothecin, a plasmid in which the human HIF-1α 5′UTR was inserted upstream of the coding sequence of EGFP was transfected into HEK293 cells. However, treatment with mitomycin C or camptothecin did not affect the EGFP protein expression (Fig. 6a). We also determined the effect of the 5′UTR in the context of the HIF-1α coding sequence using transfected wild type and P402A/P564A mutant HIF-1α (Fig. 6b,c). Mitomycin C also inhibited the expression of transfected wild type and mutant HIF-1α, while camptothecin gave variable results. However, we noted that both drugs also reduced the concentrations of other short lived transfected proteins, while not affecting the corresponding endogenous proteins (data not shown). These results suggested that the effects of the drugs on transfected proteins are at least partially due to non-specific mechanisms. Nevertheless, if mitomycin C and camptothecin regulate HIF-1α in a manner dependent on its 5′UTR, differential effects would be expected when using HIF-1α expression plasmids containing or lacking the 5′UTR. When measuring the inhibitory effects of the drugs using these plasmids, no significant differences were observed for transfected HIF-1α with or without the HIF-1α 5′UTR (see Fig. 6b and c for wild type and P402A/P564A HIF-1α, respectively). These results suggest that the inhibitory effect of mitomycin C and camptothecin on HIF-1α protein expression is independent of the 5′UTR.

In a recent report, the RNA binding proteins HuR and PTB were identified as important regulators of HIF-1α protein translation [12]. We therefore determined whether the HIF-1α inhibitors affected abundance or subcellular localization of both PTP and HuR. As shown in Supplementary Fig. S4a, mitomycin C, but not camptothecin increased the cellular HuR concentration, while none of the compounds afftected the PTB protein concentration. Given that HuR increases HIF-1α translation [12], the upregulation of HuR by mitomycin C is unlikely to account for its HIF-1α inhibitory effect. Of note, cellular stress is known to induce cytoplasmic translocation of HuR into granular structures. Thus, we also determined the effect of mitomycin C and camptothecin on HuR and PTB subcellular localization by immunofluorescence. However, we found no significant difference in cells treated with the inhibitors compared to control cells (Supplementary Fig. S4b). In both treated and untreated cells, PTB and HuR were localized to the nucleus and there was no evidence for cytoplasmic accumulation of HuR in granules. We conclude that the inhibitory effect of mitomycin C and camptothecin on HIF-1α protein synthesis is unlikely to be mediated by the RNA binding proteins HuR and PTB. However, we cannot rule out the possibility that the drugs affect binding of HuR and PTB to HIF-1α mRNA without affecting the cellular concentration or localization of the RNA binding proteins.

A major pathway through which protein synthesis is controlled is the Akt/mTOR pathway. For instance, a recently identified HIF inhibitor, KCF72, has been reported to inhibit HIF-1α protein synthesis and suppress the phosphorylation of eukaryotic translation initiation factor 4E binding protein 1 (4E-BP1) and p70 S6 kinase, two key regulators of protein synthesis and substrates of mTOR (23). We therefore determined the effect of the drugs on the Akt/mTOR pathway using phosphospecific antibodies for Akt and for mTOR substrates. Both drugs were without effect on Akt phosphorylation at Thr308 and Ser473 (Supplementary Fig. S5a). Furthermore, both mitomycin C and camptothecin did not decrease, but actually increased p70 S6 kinase (Supplementary Fig. S5b) and 4E-BP1 phosphorylation (data not shown). These results indicate that inhibition of HIF-1α protein synthesis by mitomycin C and camptothecin is independent of the Akt/mTOR pathway. Both compounds also did not suppress phosphorylation of eukaryotic initiation factor 4E (eIF-4E) at Ser209 (Supplementary Fig. S5c), which has recently been reported as a target of the HIF-1α inhibitor 6a-tigloyloxychaparrinone [42].

**Figure 7:**
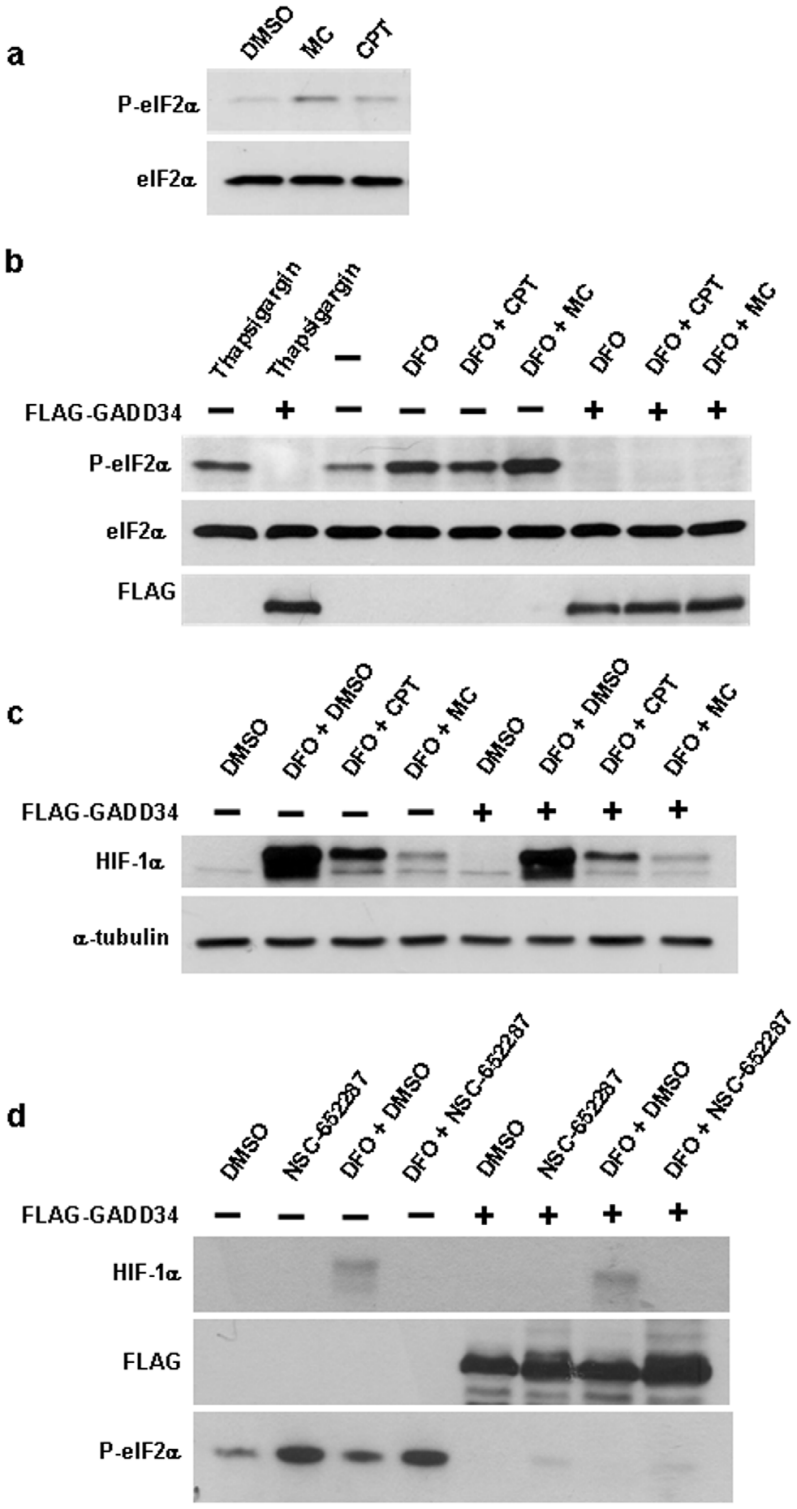
The role of eIF2α phosphorylation in the mechanism of action of HIF-1α inhibitors. (a) HEK293 cells treated with 10 µg/ml mitomycin or 2 µM camptothecin for 10 hours followed by Western blotting with antibodies specific for Ser51 phosphorylated and total eIF2α. (b–d) Cells were transfected with empty vector (control) or FLAG-GADD34 for two days followed by treatment with the indicated drugs: 1 µM thapsigargin, 200 µM desferrioxamine, 10 µg/ml mitomycin, 2 µM camptothecin, 1 µM NSC-652287. Treatment times were 10 hours. Western blotting of cell lysates was performed using the indicated antibodies.

Various cellular stress signals can induce the phosphorylation of eIF2α at Ser51, thus leading to global inhibition of cellular protein synthesis. Is has recently been reported that inhibition of the 26S proteasome or activation of a p53 dependent DNA damage response with 2,5-bis(5-hydroxymethyl-2-thienyl furan (NSC-652287, RITA) lead to specific HIF-1α translational inhibition via induction of eIF2α phosphorylation [13], [14]. Given the selectivity of the HIF-1α inhibitory effect of the drugs used in these studies, it is currently not clear, however, why the translation of other proteins is not affected. When measuring eIF2α phosphorylation at Ser51 using a phosphospecific antibody, we found that mitomycin C and, to a lesser degree, camptothecin induced phosphorylation of eIF2α without affecting its total concentrations (Fig. 7a). Of note, desferrioxamine treatment alone also resulted in marked eIF2α phosphorylation (see Fig. 7b). We also confirmed induction of phosphorylated eIF2α upon treatment with NSC-652287 (Fig. 7d). In subsequent experiments we tested whether phosphorylation of eIF2α is required for mitomycin C, camptothecin and NSC-652287 dependent inhibition of HIF-1α protein synthesis by overexpressing an active C-terminal fragment of GADD34. This protein is a substrate-specific regulatory subunit of the protein phosphatase 1 (PP1). The C-terminal GADD34 fragment is sufficient to recruit PP1 to eIF2α and cause its dephosphorylation, as previously shown in cells and *in vivo* in mice [43], [44]. Overexpression of this GADD34 construct indeed prevented basal as well as thapsigargin-induced phosphorylation of eIF2α (Fig. 7b). It also prevented mitomycin C induced phosphorylation of eIF2α (Fig. 7b). We then determined the effect of overexpressing the GADD34 plasmid on HIF-1α protein concentrations (Fig. 7c). There was no effect on basal and desferrioxamine-induced HIF-1α protein expression. GADD34 overexpression also had no effect on the inhibition of HIF-1α by mitomycin C and camptothecin. When using NSC-652287, a marked inhibitory effect on desferrioxamine induced HIF-1α expression was observed in the presence of the drug (Fig. 7d). However, this was not affected by GADD34 overexpression, despite almost complete inhibition of eIF2α phosphorylation. Taken together, these results indicate that although the DNA damage inducing agents induce the phosphorylation of eIF2α, this effect is unlikely to account for their HIF-1α inhibitory effects.

### The HIF-1α inhibitory effect of camptothecin requires cyclin-dependent kinase (Cdk) activity

**Figure 8:**
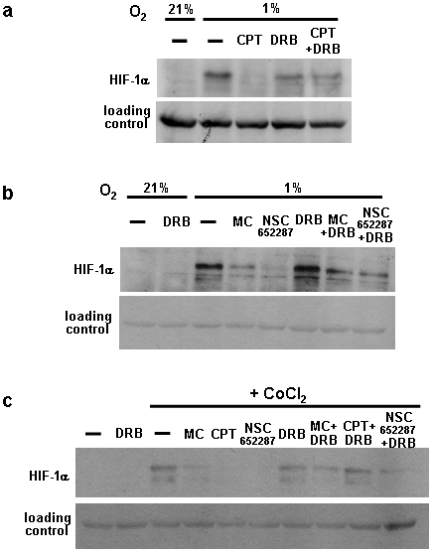
Inhibition of Cdk activity reverses HIF-1α inhibition by camptothecin. (a) HEK293 cells were treated with 2 µM camptothecin and 50 µM DRB in hypoxia for 6 hours The lysates were used for Western blotting using HIF-1α antibody. A non-specific band detected with the HIF-1α antibody served as loading control. (b,c) As in (a), the drug used concentrations were CoCl_2_ (200 µM), mitomycin C (10 µg/ml), NSC-652287 (1 µM).

A recent study reported that camptothecin triggered transcriptional stress leads to the induction of a novel transcript that is antisense to human HIF-1α mRNA [45]. The induction of the antisense transcript is dependent on topoisomerase 1 and Cdk activity, but independent of a functional DNA damage response. To determine whether this effect may account for the inhibitory effects of camptothecin on HIF-1α protein translation, we cotreated cells with camptothecin and the Cdk inhibitor 5,6-di-chloro-1-β-D-ribofuranosyl-benzimidazole (DRB). As shown in Fig. 8a, camptothecin prevented hypoxia induced HIF-1α expression, and this effect was reversed by DRB. The Cdk inhibitor also prevented the effect of camptothecin when HIF-1α was induced with CoCl_2_ (Fig. 8c). In contrast, DRB did not result in a consistent rescue of the inhibition of HIF-1α expression by mitomycin C and NSC-652287 (Fig. 8b and c). Our results provide evidence that the inhibition of HIF-1α protein synthesis by camptothecin may be the consequence of induction of a HIF-1α antisense transcript [45]. This transcript comprises the HIF-1α antisense sequence corresponding to parts of intron 1, exon 1 to several hundred bases upstream of the transcription start site [45]. It is possible that binding of the antisense transcript to the 5′end of the HIF-1α mRNA prevents efficient mRNA translation. However, it is also possible that Cdk activity regulates HIF-1α translation in response to DNA damaging agents via alternative mechanisms.

### Conclusions

HIF has emerged as an important therapeutic target in anti-cancer therapy. The oxygen sensitive HIF-1α subunit of the dimeric HIF transcription factor is primarily regulated at the level of its protein stability. However, in most cancers, normal degradation of HIF-1α is prevented, due to mutations in VHL, thus precluding HIF-1α ubiquitination by the VHL associated E3 ubiquitin ligase, or as a result of the inherently low oxygen concentrations in tumor tissue, thus preventing oxygen dependent prolyl hydroxylation of HIF-1α, which precludes binding to the VHL protein. Two reported mechanisms through which HIF-1α protein stability is regulated in an oxygen and VHL independent manner are via phosphorylation of HIF-1α by the protein kinase GSK-3 and by exposure to the DNA crosslinking agent mitomycin C [33], [34]. We show here that GSK-3 does not have universal effects on HIF-1α protein concentrations in intact cells and that mitomycin C functions by inhibiting new HIF-1α protein synthesis.

Given the difficulty to therapeutically decrease HIF-1α protein stability in tumors, most drug discovery efforts focus on inhibiting HIF transcriptional activity [e.g. ref.19]. In addition, HIF-1α protein translation has emerged as an important regulatory mechanism and a number of compounds which target HIF-1α protein synthesis have been identified. In this study we show that in addition to camptothecin, the drugs mitomycin C and YC-1 also function by inhibiting HIF-1α translation. Although the Akt/mTOR pathway and stress induced phosphorylation of eIF2α have been shown to be important regulators of HIF-1α protein synthesis rates, our data indicate that the DNA damaging agents mitomycin C and camptothecin do not inhibit HIF-1α protein expression via these pathways. Both of these pathways are global regulators of protein synthesis rates. Thus, modulation of their activity by different drugs would be unlikely to affect HIF-1α protein concentrations selectively. It has been suggested that the effect of translational inhibition is selective for HIF-1α because it is more unstable than other proteins and changes in protein synthesis rates thus become more apparent. However, we observed that other unstable proteins are not affected by mitomycin C and camptothecin treatment (see Fig. 2e). Furthermore, both drugs still inhibited HIF-1α protein expression upon inhibiting its degradation with desferrioxamine.

Our results indicate the existence of HIF-1α specific mechanisms that regulate protein translation in response to different DNA damaging drugs. These regulatory mechanisms may involve regulation by specific RNA binding proteins or elements in the 3′UTR, including microRNA binding sites and/or induction of antisense transcripts. Our results using BRCA1 knockdown (see Fig. 5c) and inhibitors of DNA damage kinases ATM, ATR and Chk1 (not shown) suggest that a functional DNA damage response is not required for drug induced inhibition of HIF-1α protein synthesis. HIF-1α translational inhibition in response to camptothecin and possibly other DNA damaging agents may be a consequence of Cdk dependent induction of a recently identified HIF-1α antisense transcript [45].

## Supporting Information

Figure S1 Effect of camptothecin and mitomycin C on general cellular protein synthesis. HEK293 cells were pretreated with 2 µM camptothecin and 10 µg/ml mitomycin C. After three hours, the medium was changed to KREBS buffer +10% fetal calf serum (and camptothecin or mitomycin C, respectively) to remove amino acids. 60 min later, 20 µCi L-[^35^S]-methionine/L-[^35^S]-cysteine Easy-Tag (PerkinElmer) was added and cells were rinsed with PBS and lysed 15, 30, and 60 min after addition of the labeled amino acids. Incorporation of L-[^35^S]-methionine/L-[^35^S]-cysteine into cellular proteins was determined by SDS-PAGE and autoradiography. In (b), 40 µM of the protein synthesis inhibitor cycloheximide was added before L-[^35^S]-methionine/L-[^35^S]-cysteine addition.
(0.13 MB TIF)

Figure S2 Dose and time dependence of mitomycin C-mediated inhibition of HIF-1α.(a) HEK293 cells were treated for 11 hours with increasing concentrations of mitomycin C (MC) in the presence of 200 µM desferrioxamine (upper panel) or under conditions of 1% oxygen (lower panel). (b) Cells were treated with 200 µM desferrioxamine. After 3 hours, mitomycin C (10 µg/ml) was added where indicated (t = 0), and cells were lysed at t = 0, 2 h, 4 h, 7 h, 10 h. A representative Western blot is shown in the left panel and a densitometry plot of the ratio of HIF-1α to α-tubulin abundance in the right panel.
(0.15 MB TIF)

Figure S3 Mitomycin C induced HIF-1α inhibition is not due to protease-dependent cleavage or accumulation of the HIF-1α protein in the triton-insoluble fraction.(a) HEK293 cells were treated with 200 µM desferrioxamine and 10 µg/ml mitomycin in the presence of 25 µM calpeptin or 10 µM pepstatin A plus 25 µM E-64 for 10 hours. (b) Cells were treated with the indicated drugs. After cell lysis, equivalent volumes of the triton X-100 soluble and insoluble fractions were separated by SDS-PAGE and analyzed with HIF-1α and α-tubulin antibodies.
(0.12 MB TIF)

Figure S4 Effect of mitomycin C and camptothecin on HuR and PTB concentrations and intracellular localization.HEK293 cells were treated with 10 µg/ml mitomycin or 2 µM camptothecin for 6 hours, followed by Western blotting or immunofluorescence staining with HuR and PTB antibodies.
(0.65 MB TIF)

Figure S5 Effect of mitomycin C and camptothecin on Akt, p70 S6 kinase and eIF-4E phosphorylation.HEK293 cells were treated with the indicated drugs (10 µg/ml mitomycin, 2 µM camptothecin, 50 µM YC-1 and 10 µM geldanamycin), followed by Western blotting with the specified Akt, p70 S6 kinase and eIF-4E antibodies.
(0.11 MB TIF)
