# Supplementary figures and images for: Inhibition of Hypoxia-Inducible Factor-1α (HIF-1α) Protein Synthesis by DNA Damage Inducing Agents
Source: PLoS One. 2010 May 7;5(5):e10522. doi: 10.1371/journal.pone.0010522 (PMC2866540; doi:10.1371/journal.pone.0010522)

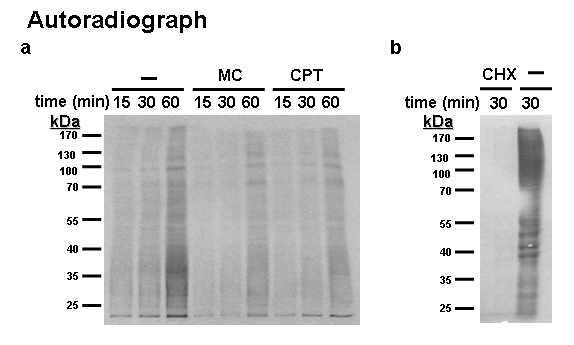

Supplement: Figure S1 — Effect of camptothecin and mitomycin C on general cellular protein synthesis. HEK293 cells were pretreated with 2 µM camptothecin and 10 µg/ml mitomycin C. After three hours, the medium was changed to KREBS buffer +10% fetal calf serum (and camptothecin or mitomycin C, respectively) to remove amino acids. 60 min later, 20 µCi L-[35S]-methionine/L-[35S]-cysteine Easy-Tag (PerkinElmer) was added and cells were rinsed with PBS and lysed 15, 30, and 60 min after addition of the labeled amino acids. Incorporation of L-[35S]-methionine/L-[35S]-cysteine into cellular proteins was determined by SDS-PAGE and autoradiography. In (b), 40 µM of the protein synthesis inhibitor cycloheximide was added before L-[35S]-methionine/L-[35S]-cysteine addition. (0.13 MB TIF) [file pone.0010522.s001.tif]

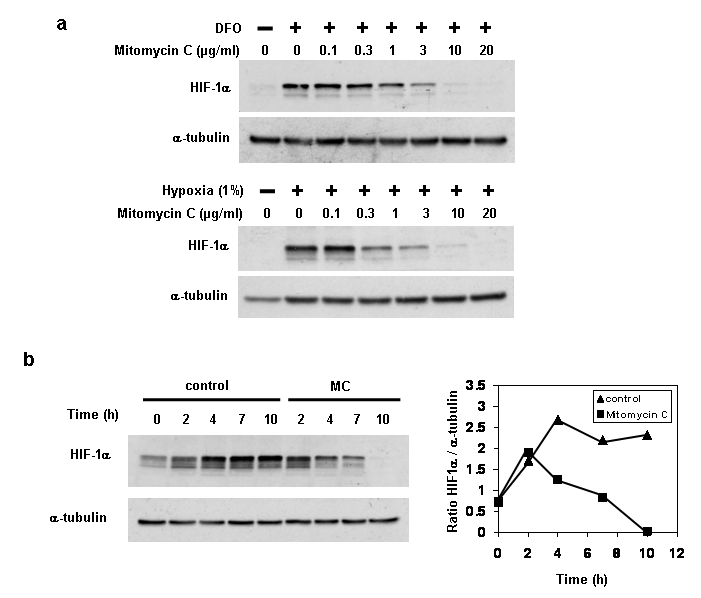

Supplement: Figure S2 — Dose and time dependence of mitomycin C-mediated inhibition of HIF-1α.(a) HEK293 cells were treated for 11 hours with increasing concentrations of mitomycin C (MC) in the presence of 200 µM desferrioxamine (upper panel) or under conditions of 1% oxygen (lower panel). (b) Cells were treated with 200 µM desferrioxamine. After 3 hours, mitomycin C (10 µg/ml) was added where indicated (t = 0), and cells were lysed at t = 0, 2 h, 4 h, 7 h, 10 h. A representative Western blot is shown in the left panel and a densitometry plot of the ratio of HIF-1α to α-tubulin abundance in the right panel. (0.15 MB TIF) [file pone.0010522.s002.tif]

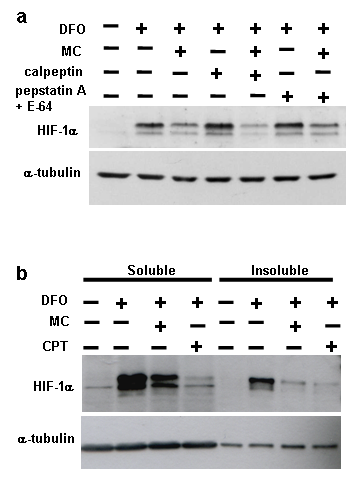

Supplement: Figure S3 — Mitomycin C induced HIF-1α inhibition is not due to protease-dependent cleavage or accumulation of the HIF-1α protein in the triton-insoluble fraction.(a) HEK293 cells were treated with 200 µM desferrioxamine and 10 µg/ml mitomycin in the presence of 25 µM calpeptin or 10 µM pepstatin A plus 25 µM E-64 for 10 hours. (b) Cells were treated with the indicated drugs. After cell lysis, equivalent volumes of the triton X-100 soluble and insoluble fractions were separated by SDS-PAGE and analyzed with HIF-1α and α-tubulin antibodies. (0.12 MB TIF) [file pone.0010522.s003.tif]

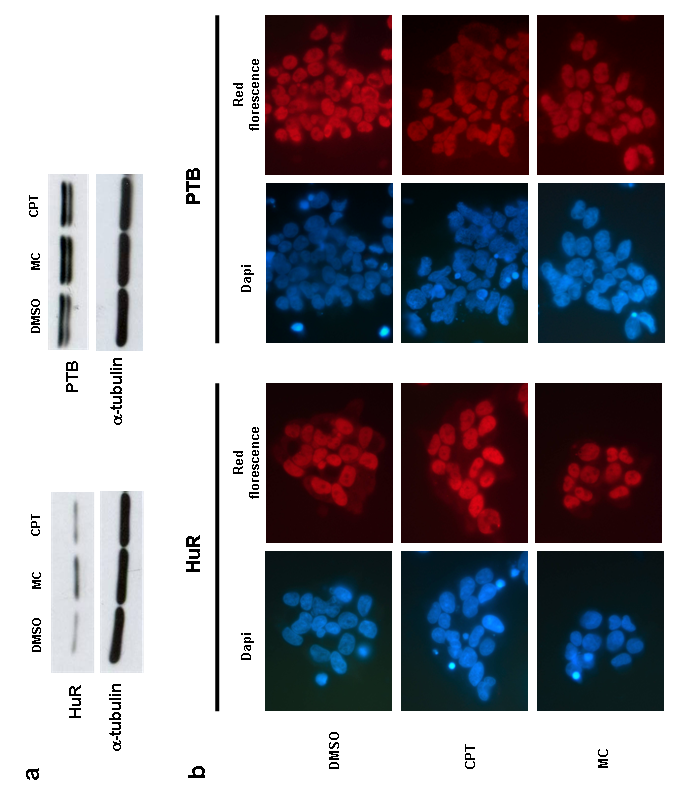

Supplement: Figure S4 — Effect of mitomycin C and camptothecin on HuR and PTB concentrations and intracellular localization.HEK293 cells were treated with 10 µg/ml mitomycin or 2 µM camptothecin for 6 hours, followed by Western blotting or immunofluorescence staining with HuR and PTB antibodies. (0.65 MB TIF) [file pone.0010522.s004.tif]

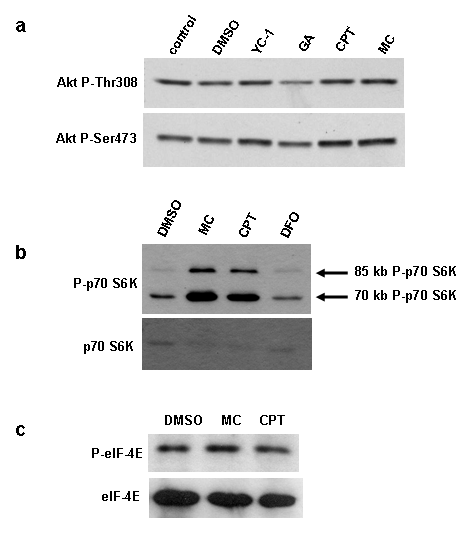

Supplement: Figure S5 — Effect of mitomycin C and camptothecin on Akt, p70 S6 kinase and eIF-4E phosphorylation.HEK293 cells were treated with the indicated drugs (10 µg/ml mitomycin, 2 µM camptothecin, 50 µM YC-1 and 10 µM geldanamycin), followed by Western blotting with the specified Akt, p70 S6 kinase and eIF-4E antibodies. (0.11 MB TIF) [file pone.0010522.s005.tif]
